# *O*-acetylated Gangliosides as Targets for Cancer Immunotherapy

**DOI:** 10.3390/cells9030741

**Published:** 2020-03-17

**Authors:** Sumeyye Cavdarli, Philippe Delannoy, Sophie Groux-Degroote

**Affiliations:** 1 UMR 8576 - UGSF - Unité de Glycobiologie Structurale et Fonctionnelle, CNRS, Université de Lille, F-59000 Lille, France; sumeyye.cavdarli@univ-lille.fr (S.C.); philippe.delannoy@univ-lille.fr (P.D.); 2 OGD2 Pharma, Institut de Recherche en Santé de l’Université de Nantes, 44007 Nantes, France; 3 Institut pour la Recherche sur le Cancer de Lille – IRCL – Place de Verdun, F-59000 Lille, France

**Keywords:** ganglioside, sialic acid, *O-*acetylation, sialate *O-*acetyltransferase, neuroectoderm derived cancer, immunotherapy

## Abstract

*O*-acetylation of sialic acid residues is one of the main modifications of gangliosides, and modulates ganglioside functions. *O*-acetylation of gangliosides is dependent on sialyl-*O*-acetyltransferases and sialyl-*O*-acetyl-esterase activities. CAS1 Domain-Containing Protein 1 (CASD1) is the only human sialyl-*O*-acetyltransferases (SOAT) described until now. *O*-acetylated ganglioside species are mainly expressed during embryonic development and in the central nervous system in healthy adults, but are re-expressed during cancer development and are considered as markers of cancers of neuroectodermal origin. However, the specific biological roles of *O*-acetylated gangliosides in developing and malignant tissues have not been extensively studied, mostly because of the requirement of specific approaches and tools for sample preparation and analysis. In this review, we summarize our current knowledge of ganglioside biosynthesis and expression in normal and pathological conditions, of ganglioside *O*-acetylation analysis and expression in cancers, and of the possible use of *O*-acetylated gangliosides as targets for cancer immunotherapy.

## 1. Introduction

Gangliosides are a sub-class of glycosphingolipids (GSL), defined by the presence of sialic acid residues. The structure of the carbohydrate moiety of gangliosides is based on a tetrasaccharide backbone substituted by up to five sialic acid residues, giving rise to a large number of oligosaccharide structures [1]. These are mostly expressed at the outer leaflet of the plasma membrane of mammalian cells where they interact with phospholipids, cholesterol, and specific transmembrane proteins, forming detergent-resistant microdomains termed “lipid rafts”. Within lipid rafts, gangliosides play an active role in regulating receptor tyrosine kinases and cell signaling pathways involved in a large number of biological processes including vesicular traffic, cell polarity, or cell proliferation [2,3]. Changes in ganglioside composition are observed in many cancers, especially from a neuroectoderm origin, which exhibit aberrant ganglioside profiles, and several ganglioside species are considered as tumor-associated carbohydrate antigens, usually associated with a poor prognosis of the patient [4]. Sialic acid residues can be also *O-*acetylated, increasing the structural heterogeneity of the oligosaccharide structures and modulating ganglioside functions. However, the effects of ganglioside *O-*acetylation are often underestimated in cancer cells due to the technical difficulties in discriminating between *O-*acetylated and non *O-*acetylated forms of gangliosides. In this review, we summarize our current knowledge on *O*-acetylated ganglioside expression patterns and evidence a potential functional significance of ganglioside *O*-acetylation in cancer. The use of *O-*acetylated gangliosides as targets for cancer immunotherapy is also discussed.

## 2. Ganglioside Structures and Biosynthesis

**Figure 1:**
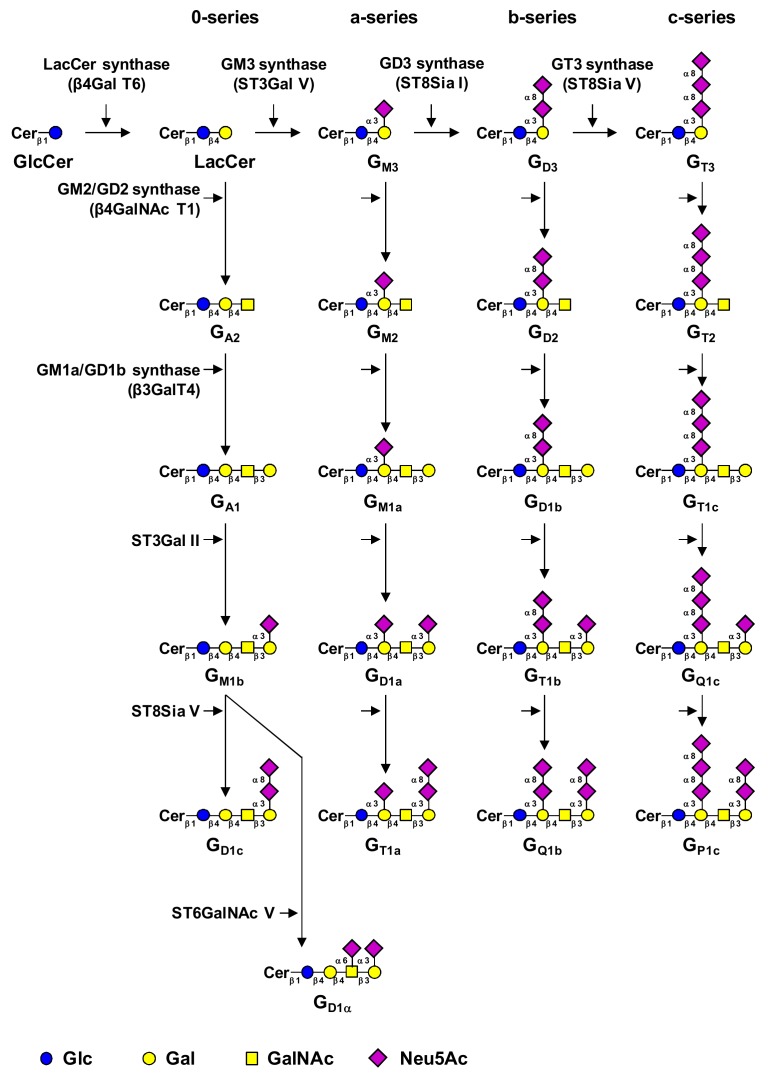
Biosynthesis of gangliosides. Gangliosides are classified in 4 series according to the number of sialic acid residues linked to lactosylceramide (LacCer) [10]. The 0-series gangliosides are directly synthesized from LacCer and the precursors of other series are synthesized by specific sialyltransferases: ST3Gal V (GM3 synthase), ST8Sia I (GD3 synthase) and ST8Sia V (GT3 synthase), respectively. The elongation of precursors is performed by the sequential action of *N*-acetylgalactosaminyltransferase (β4GalNAc T1), galactosyltransferase (β3Gal T4) and sialyltransferases (ST3Gal II and ST8Sia V). Cer, ceramide. Adapted from [3].

Gangliosides are acidic GSL containing from one to five sialic acids residues. They are mainly, but not exclusively, derived from ganglio-series GSL (GgCer) that result from the substitution of a ceramide (Cer) by the tetrasaccharide core Galβ1-3GalNAcβ1-4Galβ1-4Glcβ (Gg4). In different cell types and tissues, gangliosides are usually found as a mixture of di-, tri- and tetrasaccharide cores substituted by one or more sialic acid residues [1,5]. The biosynthesis of gangliosides takes place in the Golgi apparatus and starts by the transfer of sialic acid residues to lactosylceramide (LacCer) by specific sialyltransferases, i.e., the CMP-Neu5Ac: LacCer α2,3-sialyltransferase ST3Gal V (GM3 synthase) [6], the CMP-Neu5Ac: GM3 α2,8-sialyltransferase ST8Sia I (GD3 synthase) [7] and the CMP-Neu5Ac: GD3 α2,8-sialyltransferase ST8Sia V [8], that show high specificity toward glycolipid substrates [9]. Thus, LacCer, GM3, GD3 and GT3 are the precursors for 0-, a-, b- and c-series gangliosides, respectively (Figure 1). 

After the biosynthesis of the precursors of each series, the disaccharide core Galβ1-4Glcβ can be elongated in a stepwise manner by the transfer of *N*-acetylgalactosamine (GalNAc) and Gal residues by the β1,4-*N*-acetylgalactosaminyltransferase 1 (β4GalNAc T1, GM2/GD2 synthase) [11] and the β1,3-galactosyltransferase 4 (β3Gal T4, GM1a/GD1b synthase) [12], respectively. One or two additional sialic acid residues can be further transferred on the terminal Gal residue by the CMP-Neu5Ac: Galβ1-3GalNAc α2,3-sialyltransferase ST3Gal II [13] and the α2,8-sialyltransferase ST8Sia V. Irrespective of the elongation status of the core structure, gangliosides are characterized by the number and positions of sialic acids that define their classification in A (asialo-), M (monosialo-), D (disialo-), T (trisialo-), Q (tetrasialo-) and P (pentasialo-), and a-, b- and c series (1, 2 or 3 sialic acids on internal Gal residue, respectively) (Figure 1). Gangliosides from the 0- and a-series such as GM1a/b, GM2 or GM3 are usually considered as simple gangliosides, whereas gangliosides from b- and c-series with at least two sialic acid residues on the internal Gal such as GD3, GD2 and GD1b, are associated with complex gangliosides. In addition, the α-series is defined by the presence of a sialic acid α2,6-linked to the internal GalNAc residue, mainly transferred onto the gangliopentaosyl backbone Neu5Acα2-3Galβ1-3GalNAcβ1-4Galβ1-4Glc of GM1b by the α2,6-sialyltransferase ST6GalNAc V, to form GD1α (IV^3^Neu5Ac_1_,III^6^Neu5Ac_1_Gg_4_-Cer) (Figure 1) [14]. Although the majority of gangliosides are substituted by Neu5Ac, gangliosides containing Neu5Gc, NeuNH_2_ or their *O*-acetylated derivatives have also been reported [15,16].

The steady state of gangliosides expressed in tissues and cells depends mainly on the level of expression of glycosyltransferases (GT) involved in their biosynthesis. These GTs are typical type II membrane-anchored GTs distributed in the different cisternae of the Golgi apparatus. The first steps of ganglioside biosynthesis take place in the cis/medial-Golgi and the late steps occur in the trans-Golgi and trans-Golgi network [17]. The regulation of GT expression is mainly achieved at the transcriptional level and GT gene expression is tissue- and cell-specific. For example, the GD3 synthase ST8Sia I, the only enzyme that catalyzes the synthesis of GD3 from GM3 and controls the formation of complex gangliosides, is expressed at an early developmental stage in the fetal brain [18] and is essentially detected in the brain in adult human tissues [19]. Epigenetic regulation of ganglioside synthase genes was also described during neural development [20]. Several studies have underlined the effect of inflammation on ganglioside expression. Mice lacking TNFR1, the major receptor for TNF, demonstrated a decreased expression of GM3 and GM1b in several tissues including lung, muscle, thymus and spleen [21]. On the other hand, analysis of cell surface gangliosides in melanocytes incubated with TNF shows a higher expression of GM3 and GD3 [22]. Interestingly, TNF induces up-regulation of the GD3 synthase gene expression in ER-negative breast cancer cells via NFκB transcription factor, while in ER-positive cells, estradiol represses the TNF-induced up-regulation of GD3 synthase by inhibiting NFκB nuclear translocation [23]. Finally, GTs involved in the synthesis of gangliosides can be also regulated by post-translational modifications such as phosphorylation and dephosphorylation [24,25].

## 3. Expression of Gangliosides in Normal and Pathological Conditions

The expression patterns of monosialo- and disialo-gangliosides have been largely investigated during development and in healthy adults, as well as in numerous pathologies, such as cancers [26]. In contrast to monosialo-gangliosides that are expressed in all adult healthy tissues and are concentrated in brain and epithelial cells, complex gangliosides are highly expressed during developmental stages but exhibit low or no expression in healthy adult tissues, except in the nervous system. For example, healthy adults express GD2 and GD3 strictly in the brain, melanocytes and peripheral nerve tissues [27].

The brain is highly enriched in gangliosides compared to non-neural tissues. During brain development, the ganglioside content increases, as well as their degree of sialylation. Ganglioside expression patterns differ between species and tissues. For example, GD2, GD1b, GT1b and GQ1b are the major gangliosides in the rat central nervous system, and the expression of each ganglioside species dramatically changes during brain development [28,29]. Other data indicate changes in ganglioside content in the human brain during development; the ganglioside concentration in the human cortex exhibits a 2-fold increase between 16 and 22 weeks of gestation, mostly linked to an increase of GD1a in all regions analyzed except the cerebellar cortex, which is characterized by increasing GT1b [30]. Additionally, GD3 is ubiquitously expressed in developing mammalian healthy cerebral cortex, but shows in adult CNS a specific expression in some specific cell subpopulations, i.e., reactive astrocytes, as well as reactive and resting microglia [31].

The expression of simple gangliosides is a characteristic feature of healthy tissues. Monosialo-gangliosides GM3 and GM1 have been described to suppress the malignant properties of some cancer cells. For example, GM1 expression in tumorigenic H-ras-transformed NIH3T3 induces the suppression of proliferation [32,33]. On the contrary, disialo-gangliosides GD2 and GD3 are considered as tumor-associated carbohydrate antigens (TACA), especially in neuroectoderm-derived cancers and sarcoma [34]. GD3 and GD2 are over-expressed in osteosarcoma (OS) cell lines and in tumor tissues from patients [35]. They are also both markers of soft tissue sarcomas [36] and are over-expressed in human melanomas for which they are considered as promising targets for immunotherapy [37,38]. Disialo-gangliosides are markers in glioma [39], in small cell lung cancer (SCLC) [40] and in breast cancer (BC) [41]. In these different cancer types, the involvement of GD3 and/or GD2 in cell malignant properties has been suggested or clearly demonstrated.

Cancer-associated GSLs are not only tumor markers, but also exhibit important biological functions generated by glycolipid-enriched membrane microdomains, also named lipid rafts. The implication of specific gangliosides in the malignant properties of cancer cells is now better understood [32,42]. Raft-associated proteins, such as growth factor receptor integrins, are cis-interacting molecules with GSLs, resulting in the modulation of cell signals mediated by those receptors at the cell surface. The molecular mechanisms linking ganglioside over-expression to modifications in the behavior of cancer cells have been extremely well described in melanoma [43,44]. GD3 activates growth factor receptors that are combined with adhesion signals by integrins, resulting in the activation of downstream signaling molecules such as Akt, p130Cas and paxillin, and finally in increased growth and invasion of melanoma cells. The over-expression of GD3 synthase in U-251MG glioma cells induces the activation of Erk1/2, Akt, p130Cas, paxillin and focal adhesion kinase, increasing cell migration and invasion properties [39].

Importantly, although disialo-gangliosides are clearly identified as TACA in several cancer types, the modifications in ganglioside composition seem to be highly tumor type dependent, and monosialo-gangliosides are not necessarily down-regulated in cancers. GM3 is higher in primitive neuroectoderm tumors than in any astrocytoma type, and more expressed in glioblastoma multiformes than in both low-grade astrocytomas and anaplastic astrocytomas [45]. Interestingly, GM3 is not expressed in normal skin, but is detected in melanoma. GM3 expression extends from 60% of tumors in primary melanoma to 75% in metastatic melanoma [46]. The maintenance of monosialo-ganglioside expression in cancer cells suggests essential roles in the biology of cells, contrary to disialo-ganglioside expression. The biological roles of individual GSLs was studied in melanoma cell lines and allowed researchers to demonstrate major differences: serum-induced phosphorylation p130Cas and paxillin was increased in GD3+ cells compared to GD2+ or GM3+ cells. GD2 and GD3 both increased cell growth, but only GD3 might also be involved in invasion properties, whereas GD2 might contribute to metastasis. These results suggest that specific ganglioside species exhibit distinct roles in the malignant properties of melanoma cells by allowing a differential regulation of intracellular networks and signaling pathways [47].

Gangliosides carrying *O-*acetylated sialic acids are often found in developing tissues, especially of neuroectodermal origin, and are considered as markers in a variety of tumors [48]. *O-*acetylation of sialic acids modulates the biological functions of the carrier molecules in a variety of processes such as virus attachment, cell adhesion, and regulation of proliferation and apoptosis [49]. However, the specific biological roles of *O-*acetylated gangliosides in developing and malignant tissues have not been extensively studied, mostly because the detection and analysis of *O-*acetylated sialic acids require specific precautions for sample preparation and specific techniques and tools for analysis. The biosynthesis, analysis and functions of *O-*acetylated gangliosides in healthy and cancer tissues will be developed in the following parts of the review.

## 4. *O*-Acetylation of Sialic Acid Residues

*O*-acetylation of sialic acid residues is one of the main modifications of gangliosides. *O*-acetylated ganglioside species are known to be neo-expressed during cancer development and are considered as markers of cancers of neuroectoderm origin. The most studied species is 9-*O-*Ac-GD3, which is considered as an oncofetal marker in neuroblastoma, melanoma, or breast cancer. Other gangliosides such as GD2 or GT3 were also described to be *O-*acetylated. *O*-acetylated GD2 has recently become a target of interest for the treatment of GD2-positive cancers [50]. The expression pattern of *O*-acetylated gangliosides was studied in melanoma, neuroblastoma and breast cancer cell lines, and allowed to highlight the expression of *O*AcGM1, *O*AcGD3, *O*AcGD2, *O*AcGT2 and *O*AcGT3, with *O-*acetylation occurring mostly on the C9 position of sialic acid residues. MS/MS fragmentation studies revealed that *O*-acetylation can occur in a cell type dependent manner on both inner and terminal sialic acid residue of b- and c-series of gangliosides. These data highlight the probable existence of different *O*-acetylation pathways for gangliosides [51]. 

**Figure 2:**
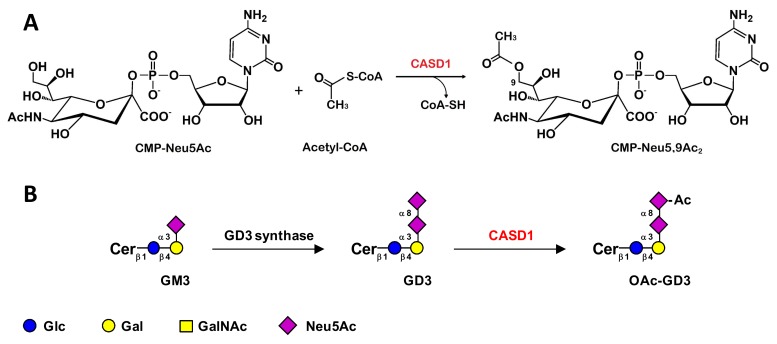
Proposed CASD1 *O*-acetyltransferase activity. (**A**) CAS1 Domain-Containing Protein 1 (CASD1) sialyl-*O*-acetyltransferases (SOAT) activity on CMP-Neu5Ac. (**B**) Biosynthesis pathway for OAcGD3 ganglioside by direct acetylation of GD3. Cer: ceramide.

The biosynthesis and degradation of *O-*acetylated gangliosides are finely regulated processes, which depend on the availability of acceptors, substrates, Golgi–ER transporters and clathrin-dependent internalization of gangliosides, and also mostly on the balance between the sialyl-*O*-acetyltransferases (SOAT) and the sialyl-*O*-acetylesterase (SOAE) activities. These finely regulated processes give rise to a tissue/cell-type specific pattern of *O*-acetylated ganglioside expression [49]. In the lumen of the Golgi apparatus, sialyltransferases first catalyze the transfer of a sialic acid residue from the activated donor CMP-sialic acid onto a Gal, GalNAc or sialic acid residue in α2-3, α2-6, or α2-8 linkage. The *O*-acetylation of sialic acid residues is catalyzed by a SOAT that transfers an acetyl group from a donor, the acetyl-coenzyme-A molecule, to the C4/7/8/9 OH-position of the sialic acid residue. The primary site of *O*-acetyl group transfer on sialic acid is thought to be C7, from which the *O*-acetyl group could subsequently migrate under acidic pH conditions to the C8 and to the C9 positions, which are more stable positions [52]. Several enzymatic activities corresponding to acetyltransferases and acetylesterases that are capable of adding or removing O-acetyl groups on different positions of sialic acids have been characterized from extracts of tissues enriched in *O-*acetylated sialic acids, with preferential acceptors for the *O*-acetylation reaction. Several acceptors, including free sialic acid, CMP-sialic acid, gangliosides, or glycoprotein-bound-sialic acid, have been shown to be substrates for SOAT, on different positions of the sialic acid residue. For example, SOAT isolated from microsomes prepared from bovine submaxillary glands preferentially transfers *O*-acetylated groups to the C7 of the sialic acid residue, which then migrate spontaneously to C8 and then to C9 due to the acidic pH, whereas the SOAT isolated from Guinea pig liver Golgi-enriched fractions transfers *O*-acetyl groups to the C4 of the sialic acid residue [53]. Regarding ganglioside *O-*acetylation, *CASD1* has been described as encoding the only human SOAT up-to-now. CASD1 (CAS1 Domain-Containing Protein 1) is ubiquitously expressed and up-regulated in human primary cell lines, melanoma and liver-derived cells. A first study in COS cells has demonstrated that the over-expression of *CASD1* and GD3 synthase genes correlates with an increase in 7-*O*AcGD3 biosynthesis, suggesting a role of CASD1 in the *O-*acetylation of gangliosides [54]. In near-human haploid HAP-1 cells, data from Baumann and coworkers suggest that CASD1 is involved in 9-*O-*acetylation of sialic acids via the formation of a covalent acetyl-enzyme intermediate, and results in increased 9-*O*AcGD3 expression. In these cellular models, CASD1 would not act directly on the GD3 ganglioside, but rather on the activated sialic acid donor, CMP-Neu5Ac [55]. In agreement with a role of CASD1 in sialic acid 9-*O-*acetylation, CASD1-deficient mice exhibit a complete loss of *O*-acetylation of Sia on the surface of hematopoietic lineage cells [56]. The biosynthesis pathways for ganglioside *O*-acetylation remain unclear, especially for species other than OAcGD3, but CASD1 seems to play a key role in the *O*-acetylation process (Figure 2).

The opposite reaction, i.e., the removal of *O*-acetyl groups from sialic acid residues, is catalyzed by a SIAE that can cleave C4- or C9-linked *O*-acetyl groups of sialic acid residues, present either on sialylated glycoconjugates, or as free sialic acids released by sialidases [57]. It is well established that the *O*-acetylation reaction takes place in the Golgi apparatus by a membrane-associated SOAT, whereas de-*O*-acetylation can either take place in the lysosomes by a membrane-bound sialyl *O*-acetyl-esterase (Lse) or in the cytoplasm by soluble esterases. The full length Lse goes though the secretory pathway and localizes primarily in lysosomes, whereas an alternative splice variant at the 5’ end encodes for a cytoplasmic SIAE. mRNA-encoding Lse are ubiquitously expressed among tissues, whereas the ones encoding cytoplasmic SIAE are mainly expressed in the liver, ovary and brain, showing the fine regulation of the sialic acid de-*O*-acetylation process [58]. Despite the limited knowledge concerning SOAT and SIAE enzymes, the diversity and differential expression of *O*-acetylated gangliosides among tissues and during development suggest regulation at multiple levels.

## 5. Analysis of *O*-Acetylated and Non-*O*-Acetylated Gangliosides Species

Many attempts have been made to profile ganglioside expression in biological samples such as blood, serum, tissues or cells, in order to identify diagnostic tools or biomarkers for diseases. Immunodetection and mass spectrometry remain the main methods used for ganglioside expression analysis. Thin Layer Chromatography (TLC) methods give an overview of ganglioside composition in different biological samples, including tissues, serum or cell pellets [59]. TLC can be based on chemical detection using orcinol but requires large amounts of material unless combined to an immunodetection. Recently, analytical techniques such as mass spectrometry combining liquid chromatography (LC-MS) or capillary gel electrophoresis have been developed for a better separation and characterization of carbohydrate and ceramide moieties of gangliosides. Fourier Transform Ion Cyclotron Mass Resonance (FT-ICR) mass spectrometry allows to separate and to distinguish the properties of sphingoid bases and fatty acid chains, while Ultra-Performance Liquid Chromatography Quadrupole Time of Flight Mass Spectrometry (UPLC-Q-TOF-MS) can be used to separate glycosphingolipid isomers with different glycan chains. For example, GD1a and GD1b can be separated and distinguished using UPLC-Q-TOF-MS [60]. In breast cancer, ganglioside profiling in sera has been performed by Liquid-Chromatography Fourier Transform Mass Spectrometry (LC-FTMS), leading to the determination of 49 species of gangliosides present in sera from patients with breast cancer, which have been compared to healthy volunteers. Li et al. identified and quantified the expression of GM1, GM2, GM3, GD3 and GT1 species with a prevalence of GM3 in sera from breast cancer patients, suggesting that GM3 could serve as a potential biomarker [61]. Sugar labeling is extensively performed for liquid chromatography analysis. Isobaric labeling of gangliosides by chemoselective oxidation of the sialic acid side chain, followed by oxydation and ligation with a carbonyl reactive tandem mass tag reagent on C9 generates fluorescent gangliosides, which can be identified and quantified using Reverse-Phase Liquid Chromatography Mass Spectrometry (RPLC-MS). RPLC-MS allows the detection and quantification of GM1a, GD1a and GT1b as major brain porcine gangliosides [62]. Rossdam and coworkers have set up a multiplex Capillary Gel Electrophoresis coupled to Laser Induced Fluorescence detection (xCGE-LIF) to analyze carbohydrate moieties of gangliosides after ceramidase digestion. This method is based on the building of a database using migration times for commercially available glycan species, and on the comparison of the retention times of biological samples to this library. xCGE-LIF allowed the characterization of GM3 and GD3 at the surface of stem-cell-derived cardiomyocytes [63]. 

All of the previously mentioned methods allow identification of the carbohydrate moiety or the ceramide moiety of gangliosides in different samples. Nevertheless, the detection and the analysis of *O*-acetylated gangliosides remain challenging due to the lability of the *O*-acetyl group using classical procedures, immunological methods or the combination of both [49,64,65]. Immunodetection including TLC, FACS or immunocytochemistry, followed by confocal microscopy, constitutes one of the main approaches for the study of gangliosides, but requires highly specific monoclonal antibodies with minimal cross-reactivity between glycans [49]. Some of the anti-ganglioside antibodies are known for recognizing the *O*-acetylated form of the targeted gangliosides. For example, anti-GD2 14.18 antibody can also recognize the *O*-acetylated GD2 species with a lower affinity compared to its preferential target GD2 [66]. Furthermore, antibodies targeting *O*-acetylated gangliosides can also cross react, such as 493D4 [67], Jones antibody [68] or A2B5 [69], targeting both OAcGD3/OAcGT3 and OAcGT2. In turn, the specificity of anti-OAcGD2 8B6 mAb has been demonstrated by several immunodetection methods, with no 8B6 binding after the removal of the *O*-acetyl group by alkali-based treatment [66,70,71,72]. Recently, gangliosides expressed by neuroectoderm-derived cancer cells have been profiled using MALDI-TOF analysis on native gangliosides. This method avoids the loss of the *O*-acetyl group and has led to the characterization of OAcGM1, OAcGD3, OAcGD2, OAcGT3 and OAcGT2 in BC, melanoma and neuroblastoma cell lines in a cell type dependent manner, suggesting that *O*-acetylated ganglioside expression depends mainly on substrate availability and GD3 synthase expression. MS/MS fragmentation of these *O*-acetylated gangliosides species showed that *O*-acetylation can occur both on the terminal and on the subterminal sialic acid of the ganglioside carbohydrate moiety [51]. To get further insight into the position of the *O*-acetyl group on the sialic acid residue, DMB-labeled sialic acid derivatives extracted from gangliosides from BC cells were analyzed by LC-ES-MS. 9-*O*-acetyl-*N*-acetyl-neuraminic acid was identified as the main *O*-acetylated sialic acid species expressed on gangliosides from BC cells [4,51]. 

## 6. *O*-Acetylated Gangliosides in Cancers

*O*-acetylated gangliosides species are mainly expressed during the developmental stage, and to a lesser extent in the skin and brain [73]. They are neo-expressed during tumorigenesis, defining them as oncofetal markers. *O*-acetylation is one of the major common modifications of sialic acid bearing gangliosides. A single report has characterized the expression of OAcGM1, OAcGD2, OAcGD3, OAcGT2 and OAcGT3 in neuroectoderm-derived cells [51]. Whereas OAcGD2 and OAcGD3 expression in cancers is very well documented, little is known concerning OAcGM1, OAcGT2 and OAcGT3. OAcGD3 expression has been reported in childhood lymphoblastic leukemia [74], glioblastoma [75], BC [76] and in basaliomas [73]. OAcGD3 has been defined as an oncofetal marker in melanoma [77]. OAcGT3 expression has been reported as a mixture of OAcGD3/OAcGT3 in the bovine brain, cultured glial cells and BC cells [67,76]. Besides this, OAcGD2 expression has been established in many neuroectoderm-derived tumor types such as neuroblastoma (NB), glioblastoma, SCLC and BC [51,71,72]. The pattern of *O*-acetylated ganglioside expression is highly tumor type dependent, suggesting a highly regulated biosynthesis mechanism and potential roles in tumor biology.

The pro-survival role of OAcGD3 has been highlighted by the upregulation of OAcGD3 in the brain. Ribeiro-Resende and coworkers reported that OAcGD3 is upregulated in regenerating peripheral nerve fibers, suggesting that OAcGD3 is involved in neuronal growth and axon regeneration [78]. Furthermore, *O*-acetylation of GD3 suppresses the pro-apoptotic role of GD3 by relocating it from the mitochondria in acute lymphoblastic leukemia [79,80]. The role of OAcGD2 in cancer cell properties has been investigated using the anti-OAcGD2 antibody 8B6. 8B6 mAb binding to OAcGD2-expressing cancer cell lines induced mitochondrial apoptosis and cell cycle arrest in vitro, and decreased tumor growth in vivo [81]. OAcGD2 is not expressed in healthy tissues, especially in peripheral nerve fibers, which renders OAcGD2 as an alternative therapeutic target to GD2 in neuroectoderm-derived tumors [71,72,81].

## 7. *O-*Acetylated Gangliosides as Targets for Immunotherapy

Cancer therapy is mainly based on surgery, radiation, chemotherapy, immunotherapy and targeted therapy. Immunotherapy is a rapidly growing field for cancer therapy, activating the immune system through active or passive immunotherapy [82]. More than 80 therapeutic antibodies have been granted permission for clinical use and more than 570 are currently being tested in various clinical phases [83]. *O*-acetylated ganglioside species represent therapeutic targets of interest as an alternative strategy to non-*O*-acetylated ganglioside species. For example, targeting OAcGD2 rather than GD2 seems to be a better strategy due to exclusive OAcGD2 expression in cancer tissue [71]. In this light, a therapeutic antibody against OAcGD2 has been developed by Cerato and coworkers as it seems a safer target than GD2. 8B6 mAb was obtained by immunizing A/J mice against NB cell lines using the hybridoma technology. Interestingly, the 8B6 mAb antibody is extremely specific for OAcGD2 [70]. In NB, anti-GD2 14.18 mAb antibody treatment induces severe toxicity side effects due to GD2 expression in healthy tissues, especially on nerve fibers. The specificity of the 8B6 antibody against OAcGD2 offers an interesting alternative to limit this toxicity, since OAcGD2 expression is limited to cancer cells. Murine 8B6 mAb shows the same efficacy as 14G2a antibody for tumor growth suppression in vitro and in vivo. 8B6 mAb induces mitochondrial-induced cell death by activation of caspase 3, leading to apoptosis of NB cell lines [81]. The chimerization of 8B6 was performed by switching murine IgG3 to human IgG1. This process induced the loss of apoptotic properties of 8B6 mAb due to the variation on the whole structure of the antibody, especially of the Fc domain. The therapeutic combination of chemotherapy supplemented by antibody targeting is another strategy preventing the recurrence of cancer diseases. 8B6 mAb has been used as an adjuvant immunotherapy in glioblastoma multiforme preventing temozolomide resistance induced by glioma stem-like cells [84]. The combination of 8B6 mAb with the chemotherapeutic agent topotecan inhibits NB cell growth and increases topotecan uptake by the activation of caspase 3, enhancing the anti-tumor efficacy of topotecan in vivo [85].

Recombinant antibodies are single-domain antibodies that have some unique features such as small size, high stability, enhanced binding properties and high penetration in dense tissues, and are used for targeting tumor-associated carbohydrate antigen [86,87]. These properties enhance the efficiency of cancer treatment by targeting the tumor environment and promoting tumor infiltration [88]. This is a growing field of antibody engineering, since their pharmacological and structural properties lead to resistance in harsh environments (such as the strong pH of the stomach), infiltration of the blood–brain barrier and reaching intracellular targets due to their small size. Recombinant antibodies include single chain variable fragments (scFv), diabodies, nanobodies, bispecific antibodies or antibody drug conjugates (ADC) [89]. ScFv is designed as a small single polypeptide constituted by the fusion of VH and VL domains via a linker peptide which can fuse to one another to generate bispecific or multi-specific antibodies [90]. Bispecific antibodies refer to a class of constructs which can recognize two antigens by their specific binding sites within one molecule by recombinant DNA or cell-fusion technologies [91]. Indeed, bispecific antibodies used in tumor immunotherapy have one arm specific for a tumor antigen and the other for the effector cells. For example, anti-CD3 x anti-GD2 bispecific antibody 3F8BiAb has been developed to target CD3 on T cells and GD2 in NB cells. 3F8BiAb shows enhanced anti-tumoral activity in vitro compared to naked 3F8 antibody [92]. The development of the anti-OAcGD2 recombinant antibody should be considered since it enhances the anti-tumor activity of the antibody. Nanobodies are single domain antibodies devoid of light chains developed by mimicking camelid antibodies. Camelids produce antibodies composed of only heavy chains and a single variable domain (VHH) as a target recognition module [88,90,93]. Three categories of nanobodies exists: naked monomeric, multimeric nanobodies, or nanobodies–peptide fusion [90]. ADC could be produced with all generations of antibodies by the fusion of cytokines, chemokines or drugs to promote the cytotoxic effect of the antibody [94]. ADC is intended to increase the efficiency of cancer treatment by directly targeting cancer cells, sparing the surrounding healthy tissues.

Regarding recent breakthroughs in immunotherapy, especially, in the field of recombinant antibody engineering, the development of specific anti-*O*-acetylated ganglioside antibodies (not only for OAcGD2 but also for other *O*-acetylated ganglioside species) is more than required for cancer therapy.

## 8. Conclusions

Gangliosides, *O*-acetylated or not, are characterized as tumor-associated carbohydrate antigens, thus representing potent therapeutic targets. The *O*-acetylation of gangliosides results from highly regulated mechanisms dependent on substrate availability and glycosyltransferase expression, giving a cell-type expression pattern. *O*-acetylated ganglioside expression is mainly associated with the pro-survival function of neuroectoderm-derived tumors. OAcGD2 seems to be a safer target than its non *O*-acetylated form, and an anti-OAcGD2 antibody has been developed for cancer therapy. In this light, recent advances in the characterization of *O*-acetylated gangliosides and their targeting by antibodies have to be promoted and extended to other cancer-associated species such as OAcGM1, OAcGD3, OAcGT3 and OAcGT2.
